# Above- and Below-Ground Carbon Storage of Hydrologically Altered Mangrove Wetlands in Puerto Rico after a Hurricane

**DOI:** 10.3390/plants10091965

**Published:** 2021-09-20

**Authors:** Lauren N. Griffiths, Elix Hernandez, Elvira Cuevas, William J. Mitsch

**Affiliations:** 1 Everglades Wetland Research Park, The Water School, Florida Gulf Coast University, 4940 Bayshore Drive, Naples, FL 34112, USA; wmitsch@fgcu.edu; 2 School of Geosciences, University of South Florida, Tampa, FL 33620, USA; 3 Department of Environmental Science, University of Puerto Rico, San Juan 00925, Puerto Rico; elix.hernandez@upr.edu; 4 Center for Applied Tropical Ecology and Conservation, University of Puerto Rico, San Juan 00925, Puerto Rico; epcuevas@gmail.com; 5 Department of Biology, University of Puerto Rico, San Juan 00925, Puerto Rico

**Keywords:** mangroves, Puerto Rico, carbon storage, hydrologic disturbance, hurricanes

## Abstract

Mangrove wetlands are important ecosystems, yet human development coupled with climate change threatens mangroves and their large carbon stores. This study seeks to understand the soil carbon dynamics in hydrologically altered mangrove swamps by studying aboveground biomass estimates and belowground soil carbon concentrations in mangrove swamps with high, medium, and low levels of disturbance in Cataño, Jobos Bay, and Vieques, Puerto Rico. All three sites were affected by hurricane María in 2017, one year prior to the study. As a result of being hit by the Saffir-Simpson category 4 hurricane, the low-disturbance site had almost no living mangroves left during sampling. There was no correlation between level of hydrologic alteration and carbon storage, rather different patterns emerged for each of the three sites. At the highly disturbed location, belowground carbon mass averaged 0.048± 0.001 g-C cm^−3^ which increased with increased aboveground biomass. At the moderately disturbed location, belowground carbon mass averaged 0.047 ± 0.003 g-C cm^−3^ and corresponded to distance from open water. At the low-disturbed location, organic carbon was consistent between all sites and inorganic carbon concentrations controlled total carbon mass which averaged 0.048 ± 0.002 g-C cm^−3^. These results suggest that mangroves are adaptive and resilient and have the potential to retain their carbon storage capacities despite hydrologic alterations, but mass carbon storage within mangrove forests can be spatially variable in hydrologically altered conditions.

## 1. Introduction

Anthropogenic carbon dioxide emissions and other greenhouse gases are a major global concern due their direct effect on global climate change [1]. The Intergovernmental Panel predicts an increase in air temperatures, changes in air and marine circulation patterns, changes in precipitation patterns, increased frequency and intensity of hurricanes and storms, larger marine waves, and sea level rise globally as well as regionally [1,2,3,4,5,6]. Caribbean Islands are more vulnerable to these changes as they are affected by complex atmospheric interactions [7]. Nature-based infrastructure in Puerto Rico and the Virgin Islands, such as coastal wetlands, span from woody saline tolerant vegetation, such as mangroves, to palustrine woody/herbaceous vegetation. They reduce or minimize eminent threats to lives and livelihoods, public infrastructure security, and human health through the resiliency of their functional capacity to provide ecosystem services: (1) carbon sequestration, (2) reduction of flood impacts; (3) filtration of contamination such as sewage and heavy metals; (4) reduction of ocean swell impacts to public and private infrastructure due to cyclonic events in and off-shore disturbances; and (5) reduction of coastal erosion, among others [8,9,10]. Wetlands play a dominant role in the global carbon cycle, sequestering an estimated 1 Pg-C yr^−1^ [6,7,11]. Wetlands have been and continue to be degraded and destroyed around the world [6,12]. Global wetland degradation has the potential to reduce carbon stores by up to 50% within the first 10 years following land use change [13].

Coastal wetlands sequester large amounts of carbon because they have high sedimentation rates, soil pores saturated with water causing hypoxic or anoxic conditions, and carbon-rich, and very productive vegetation [10,11,14,15,16]. They store a significant amount of carbon belowground in soil and belowground biomass with 84% of the carbon stored belowground [17].

Coastal wetlands dominated by mangrove communities comprise 152,361 km^2^ worldwide and over 7800 km^2^ in the Caribbean [18,19,20]. Mangroves protect coastlines from tsunamis and tropical storm damage [21]. Modelling fluid dynamics and wave force measurements of the 2004 Indonesia tsunami determined wetlands shielded the coast by absorbing wave energy resulting in 8% fewer casualties in settlements located behind wetlands compared to those that were exposed [22]. Similarly, surge-wave modeling shows that mangroves played an important role in buffering storm surge, wave activity, and water inundation during hurricane Andrew in 1992 in southeast Florida [23]. Mangroves are unique in that they rarely emit methane, making them an overall net sink of atmospheric carbon [8,14]. Sanderman et al. estimated that in 2000 mangrove substrates stored 6.4 Pg-C in the top meter of soil, ranging between 86 and 729 Mg-C ha^−1^ globally [24]. However, between 2000 and 2015, up to 122 million tons of this carbon was released due to mangrove forest loss [24]. Despite these known benefits of mangrove wetlands, human development coupled with climate change effects threaten mangroves and their large carbon stores [11,16].

Direct or basin hydrological changes are the primary threats to mangrove communities [25,26,27]. Channeling rivers for flood control which reduces flooding in the short term, but alters water flows and sediment movement, increases the rate of coastal erosion [28]. Similarly, filling in wetlands to establish urban areas eliminates spatial connection, resulting in flooding and land subsidence, mostly due to climatic events [28]. Urban, agricultural, and industrial runoff to waterways is one of the most significant contributors of nutrient inputs into coastal wetlands, thus adding an additional stressor to the ecosystem that can alter mangrove ecosystem functions [29]. Past and present hydrologic alterations have affected these wetlands, however the resiliency of mangroves to different levels of anthropogenic and non-anthropogenic disturbances needs to be explored. Additionally, it is important to further understand how hydrologic disturbances might interact with major hurricane disturbances to affect the capacity of mangroves to store carbon. Hurricane disturbance in coastal regions is frequent and increasing, therefore it is critical to evaluate the effect of pulsing high-energy disturbances (hurricanes) and pressing changes (sea-level rise) in sequestering carbon in neotropical mangrove wetlands under climate change.

The goal of this study is to understand soil carbon sequestration in hydrologically altered mangrove communities representative of different past levels of disturbance in the Caribbean Islands. This project studies aboveground biomass estimates and belowground soil carbon concentrations at mangrove forests with historic differences in land use change and land cover types–sugarcane agriculture, military use, and urban and industrial development–in Puerto Rico.

We hypothesize that: (1) sites with the least historic mangrove removal will have the most carbon stored in their soils; (2) soil inorganic carbon concentrations will negatively correlate with persistent hydrologic disturbance; and (3) soil organic carbon concentrations will have a positive correlation with aboveground biomass of mangroves.

## 2. Results

### 2.1. Mangrove Forest Community Structure

The dominant species at Cucharillas and Jobos Bay was *L. racemosa* and at Vieques was *R. mangle* (Table 1). Cucharillas only had *L. racemosa* present; Jobos Bay had all three mangrove species present with *R. mangle* 14–24%, *A. germinans* 11–100%, and *L. racemosa* 60–64% at the three sites; Vieques had only *R. mangle* species present which was identified at two of the five sites, the other three sites had no living trees present.

At Cucharillas, tree density was greatest at the site closest to the freshwater Malaria Channel (Cucharillas 1) and least at the site furthest from the freshwater source (Cucharillas 2). There were no significant differences in DBH in the trees between the two sites. At Jobos Bay, the sites in the minimally disturbed Camino del Indio (ocean-side; Jobos Bay 1 & 2) were not significantly different and highest stem density and aboveground biomass was at Jobos Bay 2 and Jobos Bay 1. The site with the lowest stem density and aboveground biomass was Jobos Bay 3, the inland, more disturbed site, which only had one individual living *A. germinans*. Only two sites at Vieques had living mangroves–Vieques 3 and 4. The only species found was *R. mangle*. Average aboveground carbon pools were greatest at Cucharillas and Jobos Bay had the least aboveground carbon pool.

### 2.2. Soil Bulk Density

**Figure 1:**
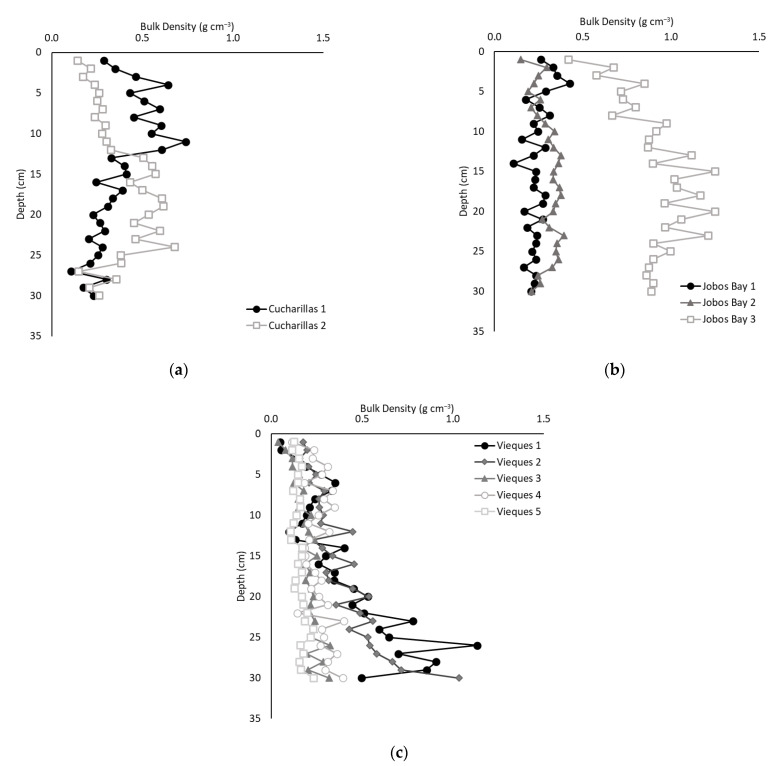
Average bulk density of soil at each 1 cm of depth at (**a**) Cucharillas, (**b**) Jobos Bay, and (**c**) Vieques.

Average bulk density ranged from 0.04 g cm^−3^ to 1.25 g cm^−3^ throughout all sites and depths (Figure 1). The average bulk densities were statistically different across all three sites with Jobos Bay the highest (0.49 ± 0.03 g cm^−3^; Figure 1b), followed by Cucharillas (0.38 ± 0.02 g cm^−3^; Figure 1a) and then Vieques (0.28 ± 0.01 g cm^−3^; Figure 1c; Kruskal-Wallis; *p* < 0.01).

At Cucharillas, there was no significant difference of bulk density between the two sites (Wilcoxon, *p* = 0.99). At Jobos Bay, Jobos Bay 1 and 2 did not have statistically different bulk density values (0.24 ± 0.06 g cm^−3^ and 0.30 ± 0.06 g cm^−3^, respectively), but Jobos Bay 3, the site furthest from the open water, which received sediments from surface flow irrigation for sugarcane production until the 1970s, had a statistically greater bulk density which averaged 0.91 ± 0.03 g cm^−3^ (Kruskal-Wallis, *p* < 0.01). At Vieques, the two sites closest to the open water (Vieques 1 and 2) had the greatest bulk density (0.41 ± 0.27 g cm^−3^ and 0.39 ± 0.04 g cm^−3^, respectively). They were not statistically different from one another (Kruskal-Wallis, *p* = 0.77), but they were significantly greater than the other three sites (Kruskal-Wallis, *p* < 0.01).

### 2.3. Soil Carbon Content

**Figure 2:**
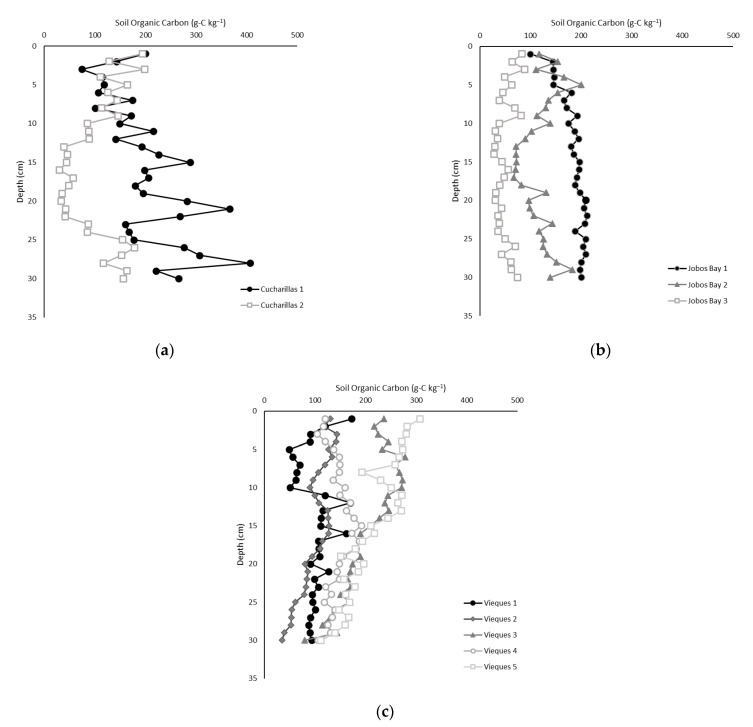
Soil organic carbon concentration at each 1 cm soil depth at each site in (**a**) Cucharillas, (**b**) Jobos Bay, and (**c**) Vieques.

**Figure 3:**
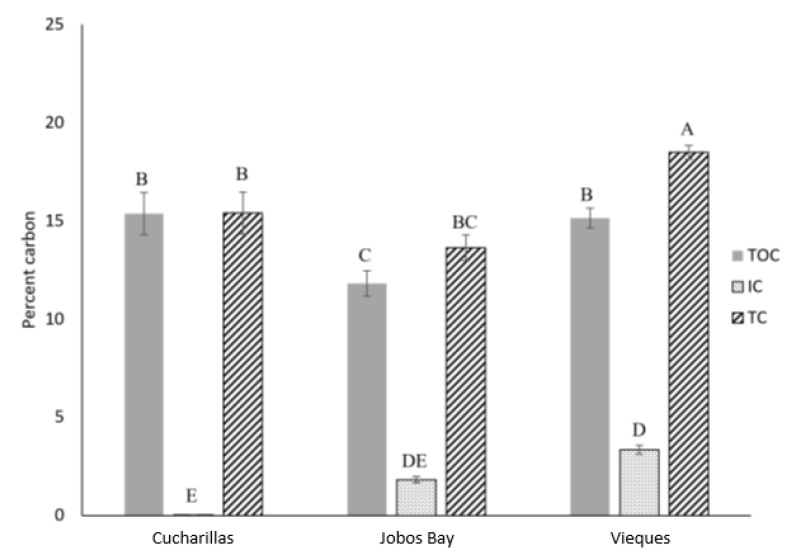
Average percent total organic carbon (TOC, shaded), inorganic carbon (IC, dotted), and total carbon (TC, striped) in the top 30 cm of soil at each location–Cucharillas, Jobos Bay, and Vieques. Error bars represent ± standard error. Similarity letters represent the results of Tukey HSD test.

Total organic carbon (TOC) content ranged from 28 to 207 g-C kg^−1^ with an average of 141.9 ± 3.9 g-C kg^−1^ across all sites and depths (Figure 2). TOC content was statistically higher at Vieques (15.1 ± 0.5%) and Cucharillas (15.4 ± 1.07%) than at Jobos Bay (11.8 ± 0.6%; Kruskal-Wallis, *p* = 0.003); however, TOC was not statistically different between Vieques and Cucharillas (Figure 3).

Percent total carbon (TC) in this study ranged from 3.1% to 40.8% and the mean was 16.4 ± 0.4%. Average TC concentrations differed significantly among the three sites (Kruskal-Wallis, *p* < 0.01); TC at Vieques averaged 18.5 ± 0.4% which was significantly greater than the TC concentrations at Jobos Bay (13.6 ± 0.6%) or Cucharillas (15.4 ± 1.1%) which were not statistically different from one another (Figure 3).

Inorganic carbon (IC) concentrations across all sites and depths ranged from 0.0024% to 9.1% and the mean was 2.2 ± 0.1%. Average IC within the cores differed significantly between the three sites (Kruskal-Wallis, *p* < 0.01). Cucharillas has the lowest IC which averaged 0.04 ± 0.01%. Jobos Bay has statistically greater IC concentrations of 1.8 ± 0.2% and Vieques has the greatest IC concentrations at 3.3 ± 0.2% (Figure 3).

**Figure 4:**
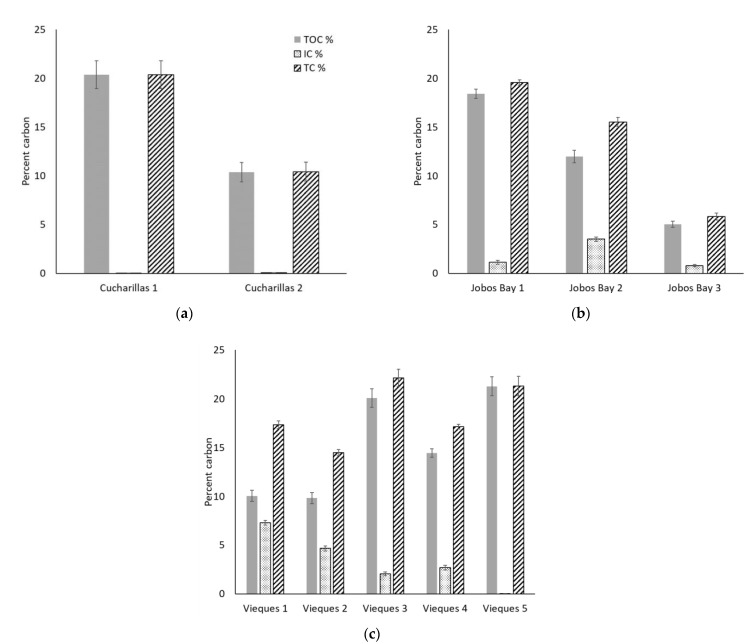
Average percent total organic carbon (TOC, shaded), inorganic carbon (IC, dotted), and total carbon (TC, striped) in the top 30 cm of soil at each site of each location–(**a**) Cucharillas, (**b**) Jobos Bay, and (**c**) Vieques. Error bars represent ± standard error.

At Cucharillas, TOC and TC decreased significantly from Cucharillas 1 (TOC: 20.4 ± 1.4%, TC: 20.4 ± 1.4%) which is closer to the open water, to Cucharillas 2 (TOC: 10.4 ± 1.0%; TC: 10.4 ± 1.0%) which is farther from the open water (Figure 4a; Wilcoxon, *p* < 0.01). IC concentrations increased with distance from open water. Cucharillas 1 has statistically lower IC concentrations (0.02 ± 0.002%) than Cucharillas 2 (0.05 ± 0.01%; Wilcoxon, *p* < 0.01).

At Jobos Bay, TOC, and TC decreased significantly at each site as the distance from open water increased (Figure 4b; Kruskal-Wallis, *p* < 0.01). Jobos Bay 1, which is closest to the open water, had the greatest TC with 19.6 ± 0.3% and TOC with 18.4 ± 0.5%. The site with the next greatest carbon concentration was Jobos Bay 2, the next closest to the open water, with 15.5 ± 0.5% TC and 12.0 ± 0.6% TOC. The lowest TC and TOC concentrations were at Jobos Bay 3, the farthest from the open water, which had 5.8 ± 0.4% TC and 5.0 ± 0.3% TOC within the soils. Inorganic carbon concentrations were statistically greater at Jobos Bay 2 (3.5 ± 0.2%) than at Jobos Bay 1 and 3 (1.1 ± 0.2% and 0.8 ± 0.01%, respectively; Kruskal-Wallis, *p* < 0.01) which were not statistically different from one another (Kruskal-Wallis, *p* = 0.41).

At Vieques, TC has significant differences between the five sites (Figure 4c; Kruskal-Wallis, *p* < 0.01). Vieques 3 and 5 have the highest total carbon concentration (22.1 ± 0.9% and 21.3 ± 1.0%, respectively) and are not statistically different from one another (Kruskal-Wallis, *p* = 0.54). Vieques 1 and 4 have the next greatest total carbon concentration (17.3 ± 0.4% and 17.1 ± 0.2%, respectively) and are not statistically different from one another (Kruskal-Wallis, *p* = 0.55). The site at Vieques with the lowest carbon concentration is Vieques 2 with 14.5 ± 0.3% total carbon which is statistically lower than all other sites (Kruskal-Wallis, *p* < 0.01). The Vieques sites also had significant differences in TOC concentrations between them (Kruskal-Wallis, *p* < 0.01). Vieques 3 and 5, like TC, have the greatest TOC concentrations (10.1 ± 0.9% and 21.3 ± 1.0%) and are not statistically different from one another (Kruskal-Wallis, *p* = 0.41). Vieques 4 has the next greatest TOC concentration with 14.5 ± 0.4% and is statistically different from all other sites (Kruskal-Wallis, *p* < 0.01). Vieques 1 and 2 have the lowest TOC concentrations (10.1 ± 0.6% and 9.8 ± 0.6%) and are not statistically different from one another (Kruskal-Wallis, *p* = 0.85). Inorganic carbon concentrations decrease with distance from open water. Vieques 1 has the greatest IC concentration with 7.3 ± 0.2% which is significantly greater than all other sites (Kruskal- Wallis, *p* < 0.01). Vieques 2 has the next statistically greatest IC concentration with 4.7 ± 0.3% (Kruskal-Wallis, *p* < 0.01). Vieques 3 and 4 have IC concentrations that are not statistically different from each other (2.1 ± 0.2% and 2.7 ± 0.3%, respectively; Kruskal-Wallis, *p* = 0.13) but are statistically greater than Vieques 5 and less than Vieques 1 and 2 (Kruskal-Wallis, *p* < 0.01). Vieques 5 has the least IC concentration with 0.03 ± 0.001% (Kruskal-Wallis, *p* < 0.01) and is the farthest site from open water.

### 2.4. Carbon Mass Soil Profile

**Figure 5:**
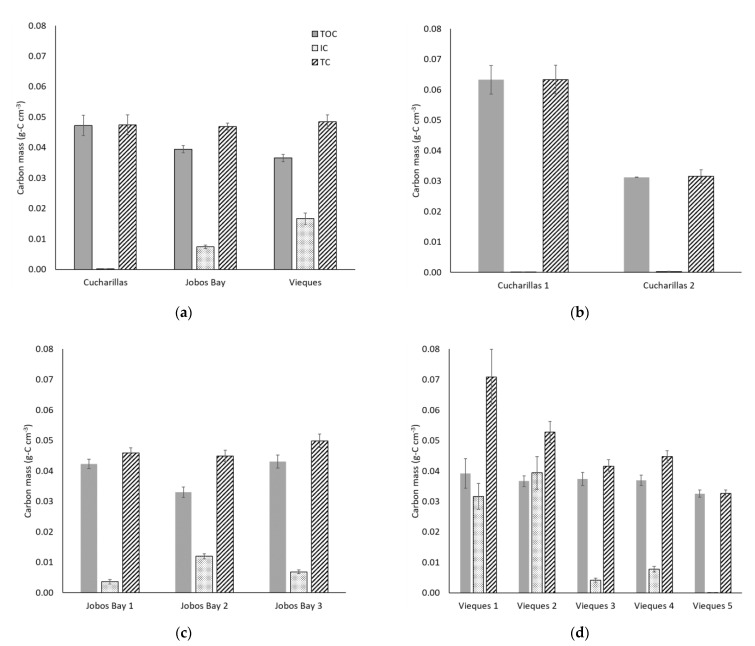
Average mass of total organic carbon (TOC, shaded), inorganic carbon (IC, dotted), and total carbon (TC, striped) per cm^3^ in the top 30 cm of soil within the sampled soil core (**a**) at each location and each site within each location–(**b**) Cucharillas, (**c**) Jobos Bay, and (**d**) Vieques. Error bars represent ± standard error.

Total organic carbon (TOC) masses range from 0.007 to 0.12 g-C cm^−3^ with an average of 0.040 ± 0.001 g-C cm^−3^ across all sites and depths (Figure 5a). TOC mass is statistically higher at Cucharillas (0.047 ± 0.003 g-C cm^−3^) and Jobos Bay (0.039 ± 0.001 g-C cm^−3^) than at Vieques (0.037 ± 0.001 g-C cm^−3^; Wilcoxon, *p* = 0.01), however TOC is not statistically different between Cucharillas and Jobos Bay (Figure 5a; Wilcoxon, *p* = 0.21).

Total carbon (TC) masses in this study range from 0.010 to 0.20 g-C cm^−3^ and the mean is 0.048 ± 0.001 g-C cm^−3^. Average TC masses do not differ significantly among the three sites (Kruskal-Wallis, *p* = 0.13); TC at Cucharillas averages 0.047 ± 0.003 g-C cm^−3^, Jobos Bay averages 0.047 ± 0.001 g-C cm^−3^, and Vieques averages 0.048 ± 0.002 g-C cm^−3^ (Figure 5a). Inorganic carbon (IC) masses across all sites and depths range from 0.000007 to 0.12 g-C cm^−3^ and the mean was 0.011 ± 0.001 g-C cm^−3^. Average IC within the cores differ significantly between the three sites (Kruskal-Wallis, *p* < 0.01). Cucharillas has the lowest IC which averaged 0.00016 ± 0.00003 g-C cm^−3^. Jobos Bay has statistically greater IC concentrations of 0.007 ± 0.001 g-C cm^−3^ and Vieques has the greatest IC concentrations at 0.017 ± 0.002 g-C cm^−3^ (Figure 5a).

At Cucharillas, TOC and TC mass decreased significantly from Cucharillas 1 (TOC: 0.063 ± 0.005 g-C cm^−3^, TC: 0.063 ± 0.005 g-C cm^−3^) which is closer to the open water, to Cucharillas 2 (TOC: 0.031 ± 0.0001 g-C cm^−3^; TC: 0.031 ± 0.002 g-C cm^−3^) which is farther from the open water (Figure 5b; Wilcoxon, *p* < 0.01). Where TOC and TC decreased with distance from the open water, IC concentrations increased with distance. Cucharillas 1 has statistically lower IC mass (0.00006 ± 0.00001 g-C cm^−3^) than Cucharillas 2 (0.00026 ± 0.00006 g-C cm^−3^; Wilcoxon, *p* < 0.01).

At Jobos Bay, TC masses had no statistical difference between sites (Figure 5c; Kruskal-Wallis, *p* = 0.27). TOC, however, significantly differed between sites (Kruskal-Wallis, *p* < 0.01). Jobos Bay 2 had statistically less TOC by mass (0.033 ± 0.002 g-C cm^−3^) than Jobos Bay 1 and 3 (0.042 ± 0.002 g-C cm^−3^ and 0.043 ± 0.002 g-C cm^−3^, respectively) which did not differ from one another (Kruskal-Wallis, *p* = 0.87). IC mass within the soils differed significantly across the three sites (Kruskal-Wallis, *p* < 0.01). Jobos Bay 2 has the greatest IC mass with 0.012 ± 0.001 g-C cm^−3^, followed Jobos Bay3 with 0.007 ± 0.001 g-C cm^−3^. Jobos Bay 1 has the least mass of carbon within the soils with 0.004 ± 0.001 g-C cm^−3^.

At Vieques, TC mass is significantly different between the five sites and decreases with distance from open water (Figure 5d; Kruskal-Wallis, *p* < 0.01). There were no statistical differences in TOC masses between the five Vieques sites (Kruskal-Wallis, *p* = 0.29). Inorganic carbon mass trends downward with distance from open water and there are statistical differences between the sites (Kruskal-Wallis, *p* < 0.01). Vieques 1 and 2 have the greatest IC masses and are not statistically different from one another (Kruskal-Wallis, *p* = 0.42). Vieques 4 has the next greatest IC mass followed closely by Vieques 3, both of which are statistically different from all other sites (Kruskal-Wallis, *p* < 0.01). By far, the lowest IC mass is at Vieques 5 which is the farthest site from open water.

**Figure 6:**
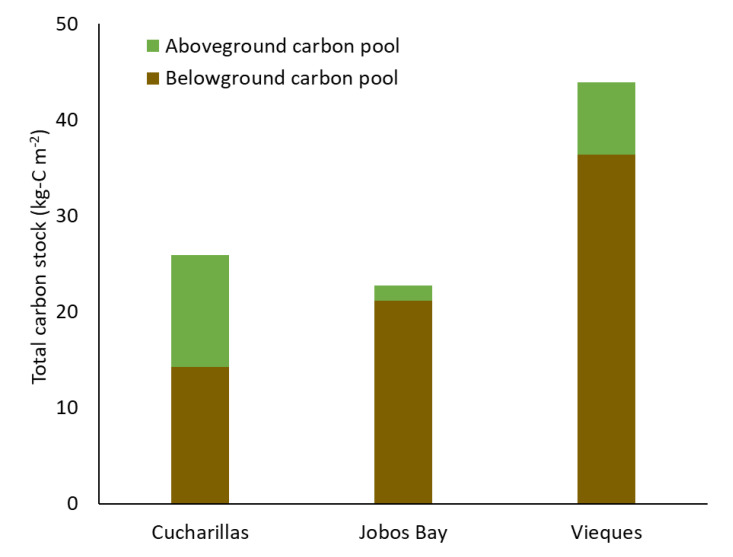
Total carbon stock in aboveground biomass (green) and belowground soil and biomass in the top 30 cm (brown) at the three study sites.

In the top 30 cm of soil, total carbon is greatest in Vieques with 36.4 kg-C m^−2^ stored in belowground biomass and soil (Figure 6). Jobos Bay has the next greatest carbon storage with approximately 21.1 kg-C m^−2^ stored belowground. Cucharillas has the least carbon stored in belowground biomass and soils with approximately 14.2 kg-C m^−2^.

## 3. Discussion

### 3.1. Mangrove Forest Community Structure

This study found that seventeen months after hurricane María, only two of the five sites sampled in Vieques had living mangroves when historically, before hurricane María in 2017, all five sites were dominated by *R. mangle* [30]. Hurricane María had devastating effects on mangroves throughout the island including defoliation and crown loss [31]. Jobos Bay exhibited hurricane effects, but epicormic growth of *A. germinans* and *L. racemosa* can cause biomass to quickly regrow after branches have been removed, trees have been knocked over, or overall tree height has been reduced whereas *R. mangle*, which was dominant at Vieques, must regrow from propagule colonization because it does not have epicormic growth when it is decapitated, so it takes a longer time to regenerate aboveground biomass [32].

At the two tidal sites at Jobos Bay, *R. mangle* is starting to take a foothold back in the wetland community with 0.12 kg m^−2^ and 0.10 stems m^−2^. Mean DBH is still small at about 2.1 cm, but overall, *R. mangle* is re-growing in these sites. Increased light availability to the forest floor caused by canopy defoliation and stem loss, can help speed up the recovery and expansion of mangrove forests after some extreme events which is evident in the Cucharillas and Jobos Bay regeneration [33]. At Vieques, however, there was large-growth *R. mangle* historically present at the sites which were completely lost to the hurricane which cannot regrow epicormically once they are decapitated, and as a result, the dense downed trees make it difficult for new propagules to take root, causing fewer of these mangroves to regenerate after hurricane María. If a forest is more densely occupied by *R. mangle*, it may be less likely for this forest to recover after a large storm because it takes too long for the mangroves to regenerate and in the meantime, redox decreases and sulfide increases to levels that are lethal to vascular plants [33,34]. Other mangrove species such as *L. racemosa* and *A. germinans* are capable of epicormic growth, causing rapid regrowth, and preventing lethal redox and sulfide levels. As a result, given the same high level of wind energy, a mangrove wetland that is predominately *R. mangle* may be less likely to fully recover from a hurricane compared to mangroves that have other species present.

Both Jobos Bay and Vieques mangrove forests are not densely occupied, and thus, aboveground biomass is more variable and not as high in these forests relative to Florida mangrove wetlands [35,36]. In Naples Bay, FL, aboveground biomass across four different sites ranged from 9.2 to 18.8 kg m^−2^ two years after a similar hurricane (hurricane Irma, September 2017) and sites were dominated by *R. mangle* [37]. In this study, across the 10 sites in 3 locations, aboveground biomass ranged from 0 to 67.1 kg m^−2^ which is a much larger range, but all sites at Jobos Bay had less aboveground biomass than the sites in Naples Bay. Scientists at Biscayne National Park in Florida also studied fringe mangroves and found aboveground biomass averaged 5.6 ± 1.2 kg m^−2^ which is lower than half of the sites studied in Puerto Rico [35]. In fringe and riverine sites around the world, aboveground biomass ranged from 0.8 to 28.7 kg m^−2^ [11] and in a meta-analysis of all mangroves around the world, aboveground biomass ranged from 0.8 to 46 kg m^−2^ [38]. Most of the sites in this study were within this range, but one site in Vieques had a much greater average aboveground biomass, with high variability (67.1 ± 28.1 kg m^−2^). It is likely that additional sampling would lower the variability and the average biomass at this site. Overall, the sites fall within the global average biomass estimates suggested in Mitsch and Gosselink [11].

### 3.2. Soil Carbon Storage

Despite other studies that have shown that hydrologic disturbance causes decreased carbon storage [16], in this study, total carbon by weight does not differ between the three sites with different levels of hydrologic disturbance. Total carbon remains the same at the three sites as a function of an increase in total organic carbon with hydrologic disturbance and an opposite but equal decrease of inorganic carbon with hydrologic disturbance. This relationship between carbon content and hydrologic disturbance supports the hypothesis that inorganic carbon will decrease with decreased hydrologic disturbance but does not support the hypothesis that less disturbed mangrove forests will store more carbon in their soils or that hydrologic restoration improves soil carbon storage.

There are different patterns observed at each of the three sites. At Cucharillas, the most hydrologically disturbed study area, soil carbon storage is greatest at the sample site with greater mangrove aboveground biomass (Cucharillas 1). Inorganic carbon is negligible at both sites, likely due to hydrologic disturbance which prevents tidal inputs and carbonate sediments as well as riverine or terrigenous inputs and siliceous sediments [39]. At the more inland site (Cucharillas 2), soil organic carbon is lower between 10 and 25 cm than throughout the rest of the core, a legacy effect of the land historically being used for development and industrial waste dumping [40]. The top 10 cm of both sites at Cucharillas have a lot of variances in soil organic carbon concentrations which is likely the result of soil mixing from the flooding and strong winds and currents that the site experienced during hurricane María.

At Jobos Bay, the moderately hydrologically disturbed site, percent TOC decreases with distance from open water, thus the sites closest to a tidal source store more organic carbon. Since the site furthest from open water has a significantly higher bulk density than the other two sites, mass of organic carbon by volume in the soil is greatest at this site despite having the lowest carbon concentration. The high bulk density at this site could be a result of the location being historically drained and used for sugarcane production whereas the other two sites have remained wetlands [41,42,43]. Despite differences in inorganic and organic carbon masses at each site, total carbon was statistically the same at all three sites at Jobos Bay. Thus, unlike at the highly hydrologically disturbed site, at Jobos Bay, which has been restored to its natural condition, there was no difference in total carbon storage between the sites. Future studies must focus on dating the soils to determine the role that hurricane María had in the inorganic and organic carbon fractions of the soil stored at both sites.

Where total carbon remains the same at all three sites in Jobos Bay, at Vieques, the least hydrologically disturbed site, total carbon mass in the soils decreases with distance from open water. Total organic carbon, however, remains consistent through all five sites and inorganic carbon decreases with distance from open water. This suggests that in a healthy, undisturbed mangrove forest, there is not a significant difference in belowground organic carbon storage within the soils, rather inorganic carbon differences control the total carbon storage. These inorganic carbon stores are likely driven by carbonate sediments brought in from tidal action, rather than terrestrial siliciclastic sediments since inorganic carbon was greatest at sites closer to the Caribbean Sea. Mangroves that are closer to the Caribbean Sea are more heavily influenced by tidal and wave action which brings in carbonate sediments that mix with the organic and siliciclastic sediments and increase overall total carbon storage [20,44]. It has been shown at other sites that hurricanes can either increase carbon storage by adding additional carbonate-rich sediments on top of the mangrove soils [45], but it is possible that these tidally influenced systems could be losing massive stores of carbon through erosion or peat collapse [20,46]. With surrounding lands having a much greater topographic relief compared to other studies, it is likely that soil erosion and carbon loss is a result of hurricanes and other large storms since studies have shown that erosion correlates with the steepness of the surrounding area [47]. Thus, it is important for further studies to focus on dating the sediments to determine the possible effects of erosion on these tidally influenced sediments during a tropical storm or hurricane.

### 3.3. Total Carbon Stock

Total carbon stock is greatest in Vieques, the undisturbed site, with approximately 43.87 kg-C m^−2^, a majority of which is stored in belowground carbon (Figure 6). Cucharillas and Jobos Bay have similar total carbon stores with 25.9 kg-C m^−2^ and 22.8 kg-C m^−2^, respectively. Although the locations have similar carbon stocks, Cucharillas has a much greater proportion of carbon stored aboveground compared to Jobos Bay. This suggests that mangrove swamps are resilient to disturbances, such as hydrologic alteration and hurricanes, because they have a backup carbon storage in above and belowground carbon storage where aboveground biomass can act as a short-term pool when soil carbon is disturbed and belowground soil and biomass act as long-term storage when aboveground biomass is decreased by acute disturbances. Mangrove forests have been shown to be resilient under natural disturbances [48]. This study supports this resilience by suggesting that above—and belowground carbon stocks provide dual carbon storage which protects the ecosystems from carbon loss after they are affected by disturbance.

### 3.4. Limitations

Limitations in this study include small sample size at some sites and spatial heterogeneity of carbon and roots across the soils sampled. At each site, only two soil cores were collected which does not gather the variability of the area and future studies should take additional cores at each site. Also, at Cucharillas, only two sites were sampled due to a lack of accessible mangroves at this location, thus conclusions from this site are not as concrete as the conclusions from Jobos Bay and Vieques. Dating of the soils using Pb-210 and Cs-137, which is ongoing, will be beneficial to further determining the role of hurricane María on these sites and help to clarify if the results are due to hydrologic differences, the hurricane, or if other factors are also affecting the carbon dynamics at these sites.

## 4. Materials and Methods

### 4.1. Study Sites

**Figure 7:**
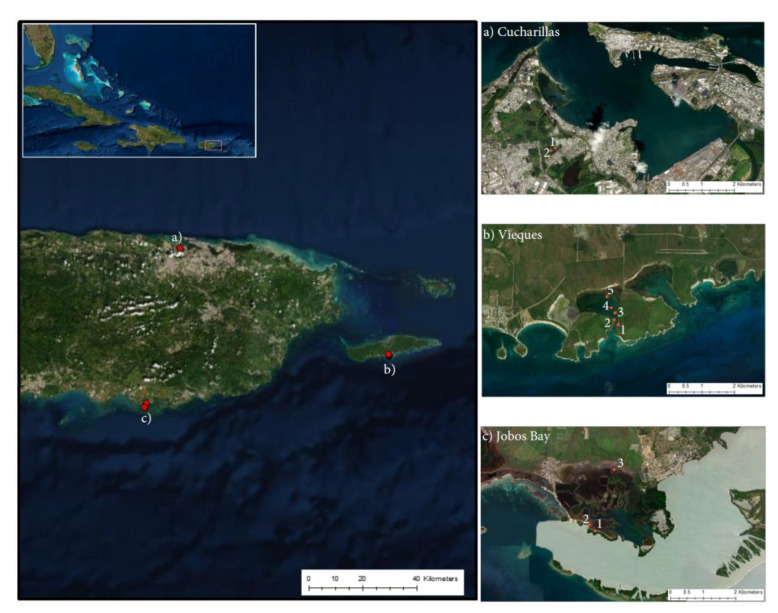
Sample sites at (**a**) Cucharillas, Cataño, (**b**) Puerto Mosquito, Vieques, and (**c**) Jobos Bay National Estuarine Research Reserve. Each sample site at each location is numbered with the lowest number closest to open, saltwater water and the largest number farthest from the marine source.

We determined soil carbon in mangrove wetlands, aboveground biomass estimates, and belowground soil carbon concentrations at mangrove forests with historic differences in land use change and land cover types–sugarcane agriculture, military use, and urban and industrial development in the archipelago of Puerto Rico, located in the northeastern portion of the Caribbean: (a) Cienaga Las Cucharillas in the western side of the San Juan Bay in the Cataño municipality, (b) Mosquito Bay in island municipality of Vieques located on the eastern side of the main island, and (c) Jobos Bay in the south eastern coast of the the main island (Figure 7). All three sites were affected by hurricane María which hit Puerto Rico as a high-end Saffir-Simpson category 4 hurricane on 20 September 2017, approximately a year and a half prior to sampling. This caused massive tree downing, decapitation and defoliation from wind and wave damage in all study sites [49]. Mosquito Bay and Jobos Bay sites have aquic soils with aridic-ustic soil moisture regimes and no direct freshwater input from rivers or streams, while the Cienaga Las Cucharillas site site has aquic soils with a typic-udic soil moisture regime [50]. The inorganic carbon component of the soils throughout Puerto Rico and at these three sites is dominated by terrigenous siliclastic sediments and marine carbonate sediments [51,52].

The Cataño study site was in Ciénaga las Cucharillas, an estuarine palustrine wetland on the western side of the San Juan Bay (18°26′11″ N, 66°8′5″ W): Cucharillas 1 is closest to the ocean (800 m inland) while Cucharillas 2 is 1 km inland [53] (Figure 7a). This site has been disturbed since Spanish colonization when channels were dredged for agricultural purposes [54]. In 1948, the Malaria Channel was dredged through the wetland linking the uppermost part of the basin to the bay. Since the 1940′s, the area has been affected by residential and industrial discharges, alterations of the basin morphology and hydrology, habitat fragmentation, leachates being dumped into the wetlands, and increased chemical pollution [40]. The site has been partially restored, but there are still major hydrologic disturbances to the area because of surrounding urbanization and industrial development [55]. The dominant vegetation of the two transects sampled was *Laguncularia racemosa (L.) C.F. Gaertn.* (white mangrove).

The sites sampled on the offshore island municipality of Vieques (Vieques 1–5, where site 1 is closest to the open ocean and site 5 is the most inland) were located in the Puerto Mosquito bioluminescent bay on the southern coast of the island (18°5″ N, 65°26″ W; Figure 7b). The mangroves at this site have remained relatively undisturbed because in the early 1940s, the U.S. Navy acquired almost 80% of the land on Vieques to use for training and ammunition supply, keeping development off the island [56]. In 2001, when the Navy left Vieques, the land was given to the U.S. Fish and Wildlife Service to be a wildlife refuge and, as such, there is 0% urban or built-up land surrounding the bay [56,57]. This site was significantly affected by hurricane María with slow regeneration.

The sites in Jobos Bay were located in the Mar Negro area within the Jobos Bay National Estuarine Research Reserve (Jobos Bay) established in 1981 (17°56″ N, 66°13″ W; Figure 7c). The inland area (Jobos Bay 3) was partially drained and used for sugar cane production until the 1970s. The ocean area (Jobos Bay1 & 2) was never used for agriculture with limited run-off freshwater inputs. Decades of subsidized freshwater inputs and fertilizer via runoff from surrounding agricultural land developed a high biomass mangrove in the inland area that suffered massive mortality when freshwater subsidies were abandoned in the 1980′s [41,42]. To the north of Jobos Bay, agricultural land dominates the watershed and increased industrial and commercial growth has been recognized as a concern [43]. Since the colonization of Puerto Rico through the 1970s, the Jobos Bay watershed was used primarily for agriculture and in the early 1900s, the expansion of agriculture led to hydrologic alterations of the landscape [43]. The hydrology of the area is determined by present semi-arid patterns of precipitation which can limit mangrove recovery [42].

### 4.2. Sampling and Data Collection

Samples were taken at 2, 3, and 5 sites at Cucharillas, Jobos Bay, and Mosquito Bay, respectively. Two soil cores were sampled at each site using a 42.24 mm diameter Russian peat corer. The corer was inserted to collect the top 1 m of soil unless rejection occurred before this point from reaching the mineral substrate. The length of each core was documented before being wrapped and transferred to a cooler where they were stored flat at 4 °C to prevent core disturbance, dehydration, and carbon transformation. Additionally, mangrove forest community structure data was collected at each site. One plot was established at each site, measuring either 4 m × 10 m or 2.5 m × 2.5 m depending on physical constraints at each site. Within each plot, species present, diameter at breast height (DBH), and stem counts were recorded for all individuals greater than 1.3 m tall. Where seedlings were present within the plots, they were counted, and their heights were recorded.

### 4.3. Laboratory Procedure

The soil cores were brought back to the lab at the University of Puerto Rico Rio Piedras where they were stored at 4 °C until the soil column was removed from the barrel, divided into 1-cm sections, and dried at 60 °C until weight was constant. All dried samples were packaged and taken to the lab at the Everglades Wetland Research Park at Florida Gulf Coast University in Naples, Florida. Dry weight was recorded for all 1-cm sections and soil samples were ground and homogenized and run through a 2-mm sieve. The homogenized mixture represents all sediments and belowground biomass from the collected core at the given depth. Bulk density was determined by using the recorded dry soil mass (M_d_) and original soil volume (V) in the following equation:

Bulk density = M_d_/V (1)

Carbon content was determined for each 1-cm segment within the top 30 cm. Only the top 30 cm of each core was analyzed because average sedimentation at Vieques and Jobos Bay is 0.2 cm yr^−1^ and in the San Juan Bay estuary ranges between 0.2–0.5 cm yr^−1^ [40,53,58,59], therefore the top 30 cm of soil represents 60–150 years of sedimentation history, capturing a complete history of hydrologic alteration and remediation at each of the sites. The 30–50 mg samples of each section were analyzed for total carbon and inorganic carbon with a Shimadzu Total Organic Carbon Analyzer (TOC-L series, SSM-5000A).

Using the DBH for each species of mangrove, aboveground biomass (W_top_; including prop roots for *Rhizophora*) was determined using allometric equations developed for each species [38,60]:

*Rhizophora mangle*   W_top_ = 0.178DBH^2.47^ (2)

*Avicennia germinans*   W_top_ = 0.0942DBH^2.54^ (3)

*Laguncularia racemose*  W_top_ = 0.209DBH^2.24^ (4)

Carbon stocks in aboveground biomass is estimated using a conversion of 45% C in dry biomass as suggested by various studies [36,37,38].

### 4.4. Statistical Analysis

Data were analyzed using JMP Pro 14 (SAS Institute Inc., Cary, NC, USA). The normality of the data was determined using the Shapiro-Wilk test. When the assumptions were not met, and data could not be transformed to meet the assumptions, non-parametric Kruskal-Wallis or Wilcoxon-signed rank test analyses were performed to determine statistical differences between sites. A *p*-value of 0.05 was used to determine statistical significance.

## 5. Conclusions

This study analyzed carbon dynamics in mangrove soils and aboveground biomass in Puerto Rico to help determine the effect of hydrologic disturbances such as hurricanes on carbon storage. We conclude the following:

- Mangroves can maintain similar function of total carbon storage despite hydrologic disturbances.
- Inorganic carbon storage appears to be negatively affected by hydrologic disturbance.
- *Rhizophora mangle* dominated mangrove swamps may be less likely to recover after an intense hurricane than mangrove wetlands supporting other mangrove species.
- Mangrove forests have adapted to hydrologic and hurricane disturbance. By allocating carbon stocks to both above and belowground pools, mangroves are able to store carbon despite potential anthropogenic or natural disturbances that may affect one of these pools.

## Figures and Tables

**Table 1:** Mangrove stem density and diameter at breast height (DBH), estimated aboveground biomass, and estimated aboveground carbon stock of mangrove species and communities from Cucharillas, Jobos Bay, and Vieques sites in Puerto Rico. Each sample site is numbered where lower numbers are sites closest to the coast and higher numbers are the most inland sites. Values are averages ± standard deviation (number of samples).

		Cucharillas		Jobos Bay				Vieques					
		Coastal	Inland	Coastal		Inland		Coastal			Inland		
		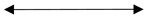		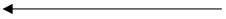				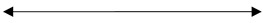					
		1	2	1	2		3	1	2	3		4	5
Tree density (stems m^−2^)	*R. mangle*	-	-	0.10	0.10		-	-	-	0.32		0.16	-
	*A. germinans*	-	-	0.05	0.18		0.03	-	-	-		-	-
	*L. racemosa*	0.15	0.08	0.28	0.43		-	-	-	-		-	-
Mean DBH (cm) ± SE	*R. mangle*	-	-	2.1 ± 0.2 (4)	2.1 ± 0.3 (4)		-	-	-	15.4 ± 9.9 (2)		13.0 (1)	-
	*A. germinans*	-	-	8.4 ± 0.9 (2)	8.0 ± 2.5 (7)		1.6 (1)	-	-	-		-	-
	*L. racemosa*	17.6 ± 4.9 (6)	29.3 ± 7.3 (2)	3.3 ± 0.3 (11)	2.1 ± 0.2 (17)		-	-	-	-		-	-
Aboveground biomass (kg m^−2^)	*R. mangle*	-	-	0.12 ± 0.01 (4)	0.12 ± 0.01 (4)		-	-	-	67.1 ± 28.1 (2)		16.1 (1)	-
	*A. germinans*	-	-	1.72 ± 0.14 (2)	7.49 ± 0.72 (7)		0.01 (1)	-	-	-		-	-
	*L. racemosa*	30.0 ± 3.0 (6)	21.8 ± 5.6 (2)	0.93 ± 0.02 (11)	0.59 ± 0.01 (17)		-	-	-	-		-	-
	Total	30.0 ± 3.0 (6)	21.8 ± 5.6 (2)	2.77 ± 0.04 (17)	8.20 ± 0.17 (28)		0.01 (1)	-	-	67.1 ± 28.1 (2)		16.1 (1)	-
Aboveground carbon stock (kg-C m^−2^)	*R. mangle*	-	-	0.06 ± 0.01 (4)	0.05 ± 0.01 (4)		-	-	-	30.2 ± 12.6 (2)		7.23 (1)	-
	*A. germinans*	-	-	0.77 ± 0.06 (2)	3.37 ± 0.32 (7)		0.003 (1)	-	-	-		-	-
	*L. racemosa*	13.5 ± 1.3 (6)	9.82 ± 2.52 (2)	0.42 ± 0.01 (11)	0.26 ± 0.01 (17)		-	-	-	-		-	-
	Total	13.5 ± 1.3 (6)	9.82 ± 2.52 (2)	1.25 ± 0.02 (17)	3.69 ± 0.08 (28)		0.003 (1)	-	-	30.2 ± 12.6 (2)		7.23 (1)	-

## Data Availability

Data is available upon request from the corresponding author.
